# A comparison of personality traits of gifted word learner and typical border collies

**DOI:** 10.1007/s10071-022-01657-x

**Published:** 2022-08-05

**Authors:** C. Fugazza, B. Turcsan, A. Sommese, S. Dror, A. Temesi, A. Miklósi

**Affiliations:** 1 Department of Ethology, Eötvös Loránd University, Budapest, Hungary; 2 Doctoral School of Biology, Institute of Biology, ELTE Eötvös Loránd University, Budapest, Hungary; 3 MTA-ELTE Comparative Ethology Research Group, Budapest, Hungary

**Keywords:** Giftedness, Personality traits, Dogs, Talent, Playfulness

## Abstract

While personality and cognition are distinct domains, some personality traits may affect the capacity for problem-solving. It was suggested that there is a positive association between the Playfulness trait and problem-solving performance in humans. Studies on giftedness (extremely good capacity in the case of a specific skill), typically aimed to reveal the genetic, experiential, and mental origins of such extreme inter-individual variation. We exploited recent findings on giftedness in a specific cognitive skill, object label learning, in dogs to explore the potential association between this exceptional skill and personality traits. We administered the Dog Personality Questionnaire to 21 gifted dog owners and compared the personality traits of their dogs to those of matched samples of 43 Hungarian and 101 Austrian typical dogs, i.e., dogs lacking this exceptional capacity. Since most Gifted Word Learner dogs are Border collies, we restricted our analysis to dogs of this breed. We hypothesized that the Gifted Word Learner dogs may show higher levels of Playfulness. As expected, we found that the gifted Border collies were rated as more playful than both the Hungarian and Austrian typical ones. Our results suggest that an extremely high level of Playfulness is associated with giftedness in a specific cognitive trait in dogs: the capacity to learn object verbal labels, thus opening new possibilities for comparative research on the relationship between giftedness and personality.

**Supplementary Information:**

The online version contains supplementary material available at 10.1007/s10071-022-01657-x.

## Introduction

Personality refers to individual differences in patterns of thinking, feeling, and behaving which are relatively stable across situations and time (Allemand et al. 2017). Similarly, there is variability in cognitive skills that allow the individual to show flexibility in solving diverse sets of problems. Thus, both personality and problem-solving skills fundamentally affect how individuals react to and interact with their environment, and thereby they contribute significantly to fitness. In humans, these traits show large inter-individual variation, and this variation has an impact on important life outcomes such as academic achievements, work success, health, and longevity (e.g. Chamorro-Premuzic and Furnham 2008; Damian et al. 2015).

While individual differences in personality traits are a widely studied topic in non-human animals, relatively little attention has been paid to individual variation in problem-solving skills as research has mostly focused on differences in the mean performance between species or experimental groups, ignoring inter-individual variation (e.g., Thornton and Lukas 2012; Boogert et al. 2018). In contrast, research in human problem-solving has recognized the significance of detecting individuals who show extreme skills. For example, Subotnik et al. (2011) refer to giftedness when the performance falls into the upper part of the distribution.

Importantly, recent research showed evidence of extreme inter-individual variation (*giftedness*) in dogs in a typical human cognitive trait: the capacity to rapidly learn multiple object labels. This skill is exceptional and manifests only in very few individual dogs within the whole population. Thus, these word learning dogs are labelled as gifted for future reference (Gifted Word Learner, GWL dogs; Fugazza et al. 2021a, 2021b). While the vast majority of dogs (typical, T dogs) struggle to learn even a few object names, the rare gifted individuals can rapidly learn multiple words without formal training, showing the ability to learn novel words in only 4 exposures (Fugazza et al. 2021a), learning at least 12 novel words in a week and remembering those for at least 2 months without practice (Dror et al. 2021). These findings paved the way for the use of these gifted dogs as models to study extreme inter-individual variation in cognitive traits (e.g., giftedness in a specific domain). Dogs are also considered one of the best model species for studying some human traits because they evolved and develop in the anthropogenic environment (Topal et al. 2009; Bunford et al. 2020); thus, they are more representative than traditional model species, such as laboratory animals.

Personality and cognition refer to functionally distinct domains, so empirical studies of these fields have typically tended to operate in isolation from one another. It has mostly been assumed that personality traits and cognitive abilities were uncorrelated (e.g. Stankov 2018) and only little effort has been made to link them. However, while cognitive abilities may be independent of personality traits, the latter may still affect problem-solving performance (i.e., the actual performance reliant on cognitive abilities).

A few authors have proposed that, in humans, some personality traits are related to problem-solving performance. For example, a meta-analysis examining the nature of the relationship between the five dimensions of personality and giftedness among individuals indicated that gifted individuals were more open to experience than non-gifted ones (Ogurlu and Özbey 2021). In humans, *Openness to Experience* is a personality trait that involves the tendency to fantasize, aesthetic sensitivity, preference for novelty, intellectual curiosity, and preference for non-traditional values (Costa Jr 1985; Costa Jr and McCrae 1992). Thus, among the Big Five personality traits, *Openness to Experience* appears to be the most conceptually proximate to *Playfulness* (Jia and Jia 2012). In humans, *Playfulness* has been found to relate to positive outcome variables such as work performance and innovative behaviour at work (Glynn and Webster 1992; Yu et al. 2007), coping (e.g., Staempfli 2007), creativity, and intrinsic motivation (Amabile et al. 1994; Proyer 2012).

Inter-individual variation in behaviour is an ecologically and evolutionarily relevant phenomenon, not only in humans but in all species. Personality studies highlighted inter-individual differences that are stable across contexts and time in a variety of behavioural characteristics such as aggressiveness, boldness, exploration, activity, and sociability in a broad range of species (e.g., Jones and Gosling 2005; Réale et al. 2010). Some studies also suggest similarities in personality traits between human and nonhuman species (Gosling 2001), including neuroendocrine correlates of personality types (Carere, et al. 2010; Koolhaas et al. 2010).

Thus, the structure of personality described for humans may overlap with that in animals (Gosling and John 1999). Moreover, positive correlations were found between owners and their dogs in all the investigated personality traits (Turcsan et al. 2012). This further endorses the applicability of similar personality models to humans and dogs.

In this study, we exploited the recent findings of extreme variation in a specific cognitive trait (giftedness in the ability to learn multiple object labels) in dogs to explore whether exceptional performance shows any association with the dogs’ personality traits.

Breed-specific differences in personality and cognition are expected to confound or mask more subtle links between the stable individual’s characteristics and their problem-solving performance. Moreover, the majority of dogs showing the exceptional skill of learning object names belong to a single breed: the Border collie, although this trait is very rare even among dogs of this breed. For these reasons, we restricted our study to Border collies.

To compare the main personality traits of the Gifted Word Learner (GWL) individuals to typical Border collies, we asked GWL dog owners to fill in a shorter version of the Dog Personality Questionnaire (DPQ-short form, developed by Jones 2008; also used in Kuroshima et al. 2016; Corrieri et al. 2018; Chopik and Weaver 2019; Posluns, et al. 2017). Due to the rarity of GWL dogs, the subjects in this group came from different countries all over the world. We then compared the personality traits of GWL Border collies to two matched samples of Hungarian and Austrian Border collies. Since the ability to learn multiple object labels is extremely rare in dogs (Fugazza et al. 2021a), we assumed that the vast majority of dogs in our comparison samples were typical Border collies, lacking this capacity. We used multiple comparison groups, including typical dogs from two different countries to better understand the variables that account for potential differences between GWL dogs’ and typical dogs’ personality traits, by disentangling those from differences that may be related to other confounding factors, such as cultural differences (Fujita et al. 2012; Horn et al. 2013; Szabó et al. 2017).

Based on the positive association between playfulness and different problem-solving skills found in human studies (Glynn and Webster 1992; Yu et al. 2007; Staempfli 2007), we hypothesized that GWL dogs would score higher on playfulness, compared to typical dogs.

## Methods

### Subjects

Three groups of Border collies (GWL dogs, Hungarian dogs, Austrian dogs), all older than 10 months of age, were included in this study. The three samples were balanced for mean age, and distribution of sex and neuter status of the dogs (i.e., a random sample has been selected from the HU and AU dogs to match the descriptives of the G dogs).

- GWL dogs: *N* = 21, mean age ± SD: 5.08 ± 2.60 years, 57.1% males, 66.7% neutered
- Hungarian: *N* = 43, mean age ± SD: 5.15 ± 3.24 years, 55.8% males, 65.1% neutered
- Austrian: *N* = 101, mean age ± SD: 5.12 ± 3.64 years, 54.5% males, 65.3% neutered

All the dogs included in the GWL dogs group knew the name of 10 > toys (Binomial test, *p* < 0.001), as tested in a baseline test carried out before this study began on all the toys available for each dog, with the methods described in (Fugazza et al. 2021a, b).

### Questionnaire

To assess dog personality, we used the Dog Personality Questionnaire (DPQ; Jones 2008). This questionnaire has been shown to demonstrate reliability and validity (Jones 2008; Posluns et al. 2017), and has been used in numerous studies to measure personality in dogs (e.g., Kuroshima et al. 2016; Corrieri et al. 2018; Chopik and Weaver 2019). The Hungarian (Ákos et al. 2014) and German translations (Riemer et al. 2016) of the questionnaire are reliable (assessed by Cronbach’s alpha) (Turcsán et al. 2018; Wallis, et al. 2020). The questionnaire was administered online. The owners were not told about the purposes of the current study, they were only informed that we were interested in the personality of their dogs.

The DPQ consisted of 45 items (S1 Table), and the owners were asked to score how much they agreed with each statement using a 5-point Likert scale. The questionnaire assessed five factors, each factor can be divided into 2 to 4 facets, and each facet is composed of three questionnaire items. The five factors were labelled as follows:

*Fearfulness* (facets: Fear of People, Nonsocial Fear, Fear of Dogs, Fear of Handling), *Aggression towards People* (facets: General Aggression, Situational Aggression), *Activity/Excitability* (facets: Excitability, Playfulness, Active Engagement, Companionability), *Responsiveness to Training* (facets: Trainability, Controllability), *Aggression towards Animals* (facets: Aggression towards Dogs, Prey Drive, Dominance over Other Dogs).

### Statistical analyses

The facet scores were calculated by averaging the scores of the raw items belonging to that facet, and the factor scores were produced by averaging the scores of the facets that made up each factor. The factor and facet scores have been transformed using square, square root, logarithmic, or cube transformation to ensure normality. However, due to the unequal sample sizes and the heterogeneity of variance in some factors (assessed by Levene’s test), we used the Welch test to compare the three samples in terms of the five factors of the DPQ. The effect size was estimated using eta squared (η^2^). When a significant difference was found in a given factor, we run additional analyses on the facets of that factor, and Games–Howell post-hoc tests (Field 2013) were run where significant differences were found. We used Cohen’s d to estimate the effect size for these pairwise comparisons. To control for the false discovery rate (FDR), we used Benjamini–Hochberg procedure (Benjamini and Hochberg 1995) to adjust the p values for multiple comparisons. SPSS (version 28, IBM Corporation) was used for all statistical analyses except for Cohen’s d which was calculated manually.

## Results

**Fig. 1:**
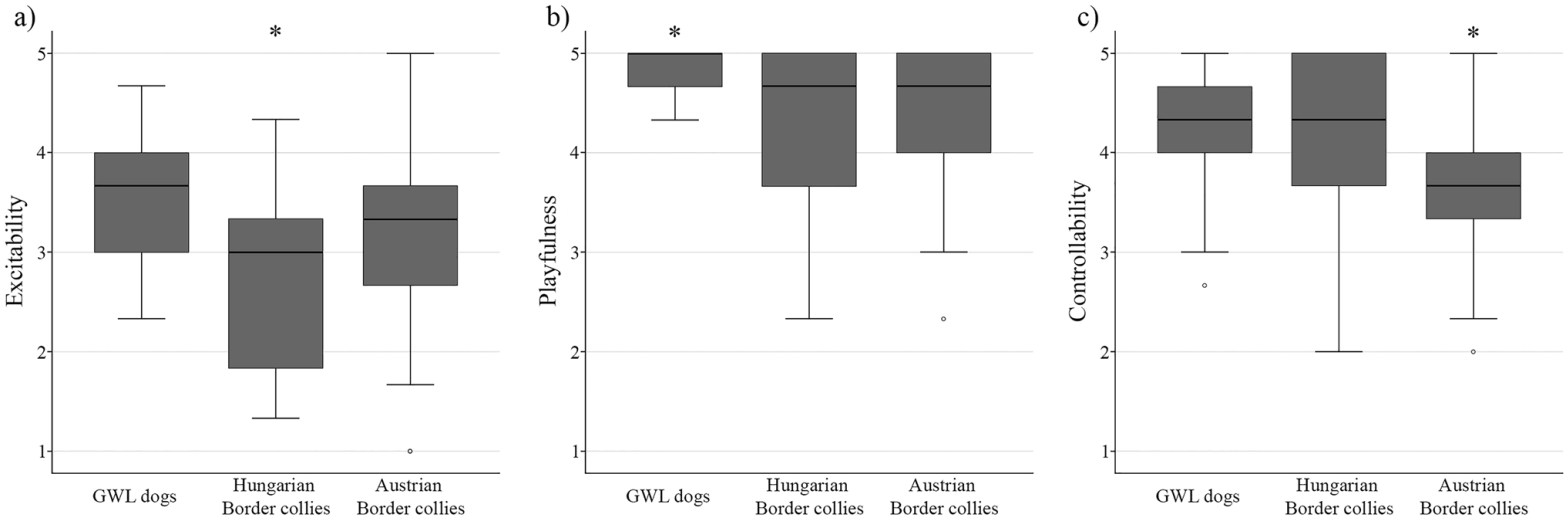
**a** Differences between the three samples of Border collies (GWL dogs, Hungarian, Austrian) in the Excitability facet of the Activity/Excitability factor. Hungarian Border collies were found to be less excitable than both the GWL dogs and the Austrian Border collies (*p* < 0.001, *p* = 0.005, respectively). **b** Differences between the three samples of Border collies (GWL dogs, Hungarian, Austrian) in the Playfulness facet of the Activity/Excitability factor. GWL dogs were found to be more playful than both the Hungarian and Austrian Border collies (*p* < 0.001 for both). **c** Differences between the three samples of Border collies (GWL dogs, Hungarian, Austrian) in the Controllability facet of the Responsiveness to Training factor. Austrian Border collies were found to be less controllable than both the GWL dogs and the Hungarian Border collies (*p* = 0.003, *p* = 0.006, respectively)

Descriptives of the means and standard deviations of the three dog groups can be found in Table 1. Fearfulness, Aggression towards People, and Aggression towards Animals did not differ significantly between the three samples of Border collies (*p* > 0.184 for all). However, we found a significant difference between the samples in Activity/Excitability (*F*_2.52.61_ = 5.137, *p* = 0.020, *η*^2^ = 0.059) and Responsiveness to training factors (*F*_2.46.21_ = 7.988, *p* = 0.003, *η*^2^ = 0.094). In the former (Activity/Excitability) factor, Hungarian Border collies received lower scores than GWL dogs (*p* = 0.020, *d* = 0.792) and tended to receive lower scores than Austrian Border collies (*p* = 0.066, *d* = 0.459). Subsequent analyses of the facets of this factor showed significant differences in Excitability and Playfulness between the samples. Regarding the Excitability facet (*F*_2.56.12_ = 10.618, *p* < 0.001, *η*^2^ = 0.104), Hungarian Border collies were found to be less excitable than both the GWL dogs (*p* < 0.001, *d* = 1.134) and the Austrian Border collies (*p* = 0.005, *d* = 0.642) (Fig. 1a). Regarding the Playfulness facet (*F*_2.69.23_ = 15.128, *p* < 0.001, *η*^2^ = 0.067), GWL dogs were more playful than both Hungarian (*d* = 1.006) and Austrian Border collies (*d* = 0.892, *p* < 0.001 for both) (Fig. 1b). The other two facets (Active Engagement, Companionability) did not differ significantly between the samples (*p* > 0.193 for both).

**Table 1:** Means and standard deviations of the Dog Personality Questionnaire factor scores in the three dog groups

Factors and facets	GWL Border collies (*N* = 21)		Hungarian Border collies (*N* = 43)		Austrian Border collies (*N* = 101)	
	Mean SD		Mean SD		Mean SD	
Factor 1—fearfulness	2.345	0.584	2.370	0.672	2.170	0.586
Facet 1—fear of people	2.349	0.703	2.124	0.861	2.178	0.855
Facet 2—nonsocial fear	2.540	0.763	2.326	0.868	2.333	0.855
Facet 3—fear of dogs	2.254	0.919	2.109	0.925	2.036	0.718
Facet 4—fear of handling	2.239	0.831	2.922	0.948	2.132	0.807
Factor 2—aggression towards people	1.857	0.740	1.605	0.664	1.612	0.613
Facet 1—general aggression	1.937	0.743	1.736	0.828	1.677	0.737
Facet 2—situational aggression	1.778	0.891	1.473	0.601	1.548	0.627
Factor 3—activity/excitability	4.218	0.373	3.895	0.440	4.093	0.422
Facet 1—excitability	3.556	0.661	2.698	0.841	3.248	0.870
Facet 2—playfulness	4.825	0.227	4.310	0.688	4.426	0.591
Facet 3—active engagement	4.460	0.543	4.337	0.613	4.337	0.538
Facet 4—companionability	4.032	0.722	4.248	0.724	4.363	0.640
Factor 4—responsiveness to training	4.254	0.593	4.078	0.822	3.787	0.531
Facet 1—trainability	4.238	0.676	3.992	0.927	3.941	0.584
Facet 2—controllability	4.270	0.655	4.163	0.886	3.634	0.662
Factor 5—aggression towards animals	2.640	0.676	2.456	0.573	2.328	0.703
Facet 1—aggression towards dogs	2.635	0.977	2.267	0.974	2.310	0.986
Facet 2—prey drive	2.587	1.000	2.287	0.884	2.201	0.885
Facet 3—dominance over other dogs	2.698	0.823	2.806	0.877	2.472	0.942

In the case of the Responsiveness to training factor, Austrian Border collies received lower scores than GWL dogs (*p* = 0.014, *d* = 0.829) and, on a trend level, also lower than Hungarian Border collies (*p* = 0.056, *d* = 0.419). Regarding the two facets of this factor, the results did not show a significant difference between the samples in Trainability (*p* = 0.193), only in Controllability (*F*_2.49.37_ = 11.986, *p* < 0.001, *η*^2^ = 0.129). In this facet, we found that Austrian Border collies received lower scores than both GWL dogs (*p* = 0.003, *d* = 0.965) and Hungarian Border collies (*p* = 0.006, *d* = 0.676) (Fig. 1c).

## Discussion

Similar to human studies (E.g., Stankov 2018; Wirthwein et al. 2019), our exploratory study did not find differences in most personality traits between the gifted and the typical dogs. The only trait that presented a significant difference between GWL Border collies and both the Austrian and Hungarian samples of typical Border collies was *Playfulness*. Thus, as hypothesized, giftedness in solving a specific problem (recognizing objects based on verbal labels) may be associated with higher levels of playfulness.

The rapid learning of toy names was shown to occur in playful social contexts with the owner (Fugazza et al. 2021a, b), which may further favour learning in very playful individuals by providing more occasions for learning. More playful dogs were also found to establish eye contact with humans faster (Bognár 2021), which may further facilitate a communicative context, such as the one in which object labels are successfully learned by GWL dogs.

Importantly, the positive association between *Playfulness* and giftedness in object label learning could emerge in two rather different ways, which are not necessarily mutually exclusive. First, some individual dogs may have a strong predisposition to play with humans, and this could result in exaggerated playful interaction within some owner–dog dyads when the owners further facilitate the existing high level of play. Alternatively, some owners living with Border collies may intently force playful activity on these dogs, assuming that this is the way how this breed should be socialized. In the first situation, playful behaviour would have some genetic basis and may be further enhanced by interaction with humans, while in the case of the latter, the exaggerated play could develop as a habitual behaviour because of environmental factors (the role of the owner). In this latter case, there may be a preponderant role of lived experiences (Bard et al. 2021; Leavens et al. 2019). Thus, a causal relationship between *Playfulness* and giftedness should not be assumed.

The Border collie is a working breed selected for herding purposes. Selection for working dog use has been shown to positively correlate with *Playfulness* (Svartberg 2006) and working dogs show more interest in playing with humans. For example, by the means of a questionnaire study, Asp et al. (2015) found that working dogs showed 30% more interest in playing with humans, compared to dogs belonging to non-working breeds. The Border collie belongs to the most playful breeds, along with other working dogs. Gifted Word Learner dogs are very rare, even among working dog breeds, thus high Playfulness, which may be a prerequisite, in itself does not explain the emergence of any specific skill. However, the GWL Border collies scored even higher than the typical ones in Playfulness. Thus, *extremely high* levels of *Playfulness* may somehow support the emergence of the capacity to learn object names.

Interestingly, it has been shown that human-directed play behaviour could have been an important trait during dog domestication (Kolm et al. 2020), and selection for particularly playful individuals may have played an important role in the later artificial selection regime that the domestic dog has gone through in the past few hundred years (Bradshaw et al. 2015). Domestication has also been shown to extend the duration of the sensitive period of socialization (Belayev et al. 1985). It could be speculated that this, in turn, may also prolong the time when flexibility in learning about specific stimuli—such as words—is maximized, thus allowing word learning in extremely playful, gifted individuals to emerge.

Our analysis also revealed some differences between personality traits in samples from the two different countries, Austria and Hungary. Hungarian Border collies were found to be less excitable than both the GWL dogs and the Austrian Border collies and Austrian Border collies received lower scores than both GWL dogs and Hungarian Border collies in Controllability. Cultural differences may explain these results. Other studies found minor differences in cognitive–behavioural tests between dogs of different countries (Fujita et al. 2012; Horn et al. 2013; Szabó et al. 2017) and cultural differences have been suggested to play a role in the similarity of personality traits between dogs and their owners (Turcsán et al. 2012). These differences may be driven by potential cultural differences in factors like dog-keeping practices, dogs’ role in the family, shared activities, and other factors affecting dog choice. It should also not be excluded that, since purebred dogs are genetically isolated populations, a further level of isolation may come from a reduced frequency of mating between Hungarian and Austrian Border collie populations, compared to within-country mating, resulting in some minor differences in the frequency of some personality traits such as the ones reported in this study (see also Wan et al. 2013).

The differences found between the three samples of dogs also highlight the importance of multiple group comparisons to understand the variables that account for differences. For example, if we hadincluded only one group of typical Border collies as a comparison, we would have obtained results indicating differences in other factors too (e.g. *Excitability* or *Controllability*). The multiple group comparison allowed us to disentangle differences that are most likely accounted for by cultural differences rather than being related to the exceptional capacity of learning object verbal labels.

Questionnaire-based studies may suffer from owner bias when they report on the behaviour of their dog (Mirkó, et al. 2013). Thus, one potential limitation of this study is that personality traits, including *Playfulness*, are based on the owner’s judgment of their dogs’ behaviour. It should also be noted that, since the learning of object labels occurs during playful interactions, once the owners notice that their dog has learned some object labels, they may start to engage in this playful activity more. This may also influence their judgment of *Playfulness* in their dog.

In summary, exaggerated *Playfulness* in dogs and frequent and intensive playful interaction with the owners may support learning object labels, but this does not explain the exceptional performance of these dogs. Further, the successful learning of object labels may have positive feedback and encourage the owner and the dog to play this, even more, this retrieval game. Importantly, we do not claim that there is a causal relationship between exaggerated *Playfulness* and general problem-solving skills in dogs because, to the best of our current knowledge, GWL dogs excel only in this specific cognitive skill.

## Supplementary Information

Below is the link to the electronic supplementary material.

Supplementary file1 (DOCX 20 KB)
