# Supplementary material for: A comparison of personality traits of gifted word learner and typical border collies
Source: Anim Cogn. 2022 Aug 5;25(6):1645–52. doi: 10.1007/s10071-022-01657-x (PMC9652199; doi:10.1007/s10071-022-01657-x)
Supplement: Supplementary file 1 — Supplementary file1 (DOCX 20 KB) [file 10071_2022_1657_MOESM1_ESM.docx]

**S1 Table** The questionnaire used in the study (DPQ ‘short form, Jones, 2008). The questions were rated on a 5-point Likert scale. The numbers before the questions indicate the order they were administered. Reverse-coded items are marked with an asterisk.

| **Factor 1 – Fearfulness** | | |  |
| --- | --- | --- | --- |
|  | **Facet 1 –** **Fear of People** | | |
|  | 1* | Dog is relaxed when greeting people. | |
|  | 6 | Dog is shy. | |
|  | 27 | Dog behaves fearfully towards unfamiliar people. | |
|  | **Facet 2 – Non-social Fear** | | |
|  | 3 | Dog is anxious. | |
|  | 11* | Dog is confident. | |
|  | 22* | Dog adapts easily to new situations and environments. | |
|  | **Facet 3 – Fear of Dogs** | | |
|  | 13 | Dog avoids other dogs. | |
|  | 21 | Dog behaves submissively (e.g., rolls over, avoids eye contact, licks lips) when greeting other dogs. | |
|  | 42 | Dog behaves fearfully towards other dogs. | |
|  | **Facet 4 – Fear of Handling** | | |
|  | 16 | Dog behaves fearfully during visits to the veterinarian. | |
|  | 35 | Dog exhibits fearful behaviours when restrained. | |
|  | 44 | Dog behaves fearfully when groomed (e.g., nails trimmed, brushed, bathed, ears cleaned). | |
| **Factor 2 – Aggression towards People** | | |  |
|  | **Facet 1 – General Aggression** | | |
|  | 7 | Dog behaves aggressively towards unfamiliar people. | |
|  | 18* | Dog is friendly towards unfamiliar people. | |
|  | 40 | Dog shows aggression when nervous or fearful. | |
|  | **Facet 2 – Situational Aggression** | | |
|  | 25 | Dog behaves aggressively in response to perceived threats from people (e.g., being cornered, having a collar reached for). | |
|  | 30 | Dog behaves aggressively during visits to the veterinarian. | |
|  | 36 | Dog aggressively guards coveted items (e.g., stolen items, treats, food bowl). | |
| **Factor 3 – Activity/Excitability** | | |  |
|  | **Facet 1 – Excitability** | | |
|  | 15 | Dog is boisterous. | |
|  | 31 | Dog seeks constant activity. | |
|  | 41* | Dog tends to be calm. | |
|  | **Facet 2 – Playfulness** | | |
|  | 9* | Dog gets bored in play quickly. | |
|  | 17 | Dog enjoys playing with toys. | |
|  | 33 | Dog retrieves objects (e.g., balls, toys, sticks). | |
|  | **Facet 3 – Active Engagement** | | |
|  | 4* | Dog is lethargic | |
|  | 14 | Dog works at tasks (e.g., getting treats out of a Kong, shredding toys) until finished. | |
|  | 24 | Dog is curious. | |
|  | **Facet 4 – Companionability** | | |
|  | 20 | Dog seeks companionship from people. | |
|  | 26* | Dog is aloof. | |
|  | 37 | Dog is affectionate. | |
| **Factor 4 – Responsiveness to Training** | | |  |
|  | **Facet 1 – Trainability** | | |
|  | 29* | Dog is slow to respond to corrections. | |
|  | 38* | Dog ignores commands. | |
|  | 43 | Dog can focus on a task in a distracting situation (e.g., in loud or busy places, around other dogs). | |
|  | **Facet 2 – Controllability** | | |
|  | 5 | When off-leash, dog comes immediately when called. | |
|  | 10* | Dog is quick to sneak out through open doors, and gates. | |
|  | 32 | Dog leaves food or objects alone when told to do so. | |
| **Factor 5 – Aggression towards Animals** | | |  |
|  | **Facet 1 – Aggression towards Dogs** | | |
|  | 2 | Dog behaves aggressively toward dogs. | |
|  | 19* | Dog is playful with other dogs. | |
|  | 34* | Dog is friendly towards other dogs. | |
|  | **Facet 2 – Prey Drive** | | |
|  | 8 | Dog likes to chase squirrels, birds, or other small animals. | |
|  | 23 | Dog likes to chase bicycles, joggers, and skateboarders. | |
|  | 39 | Dog behaves aggressively towards cats. | |
|  | **Facet 3 – Dominance over other Dogs** | | |
|  | 12 | Dog is dominant over other dogs. | |
|  | 28* | Dog willingly shares toys with other dogs. | |
|  | 45 | Dog is assertive or pushy with other dogs (e.g., if in a home with other dogs, when greeting). | |
